# ^18^F-FDOPA PET/CT Combined with MRI for Gross Tumor Volume Delineation in Patients with Skull Base Paraganglioma

**DOI:** 10.3390/cancers11010054

**Published:** 2019-01-08

**Authors:** Mehdi Helali, Matthieu Moreau, Clara Le Fèvre, Céline Heimburger, Caroline Bund, Bernard Goichot, Francis Veillon, Fabrice Hubelé, Anne Charpiot, Georges Noel, Alessio Imperiale

**Affiliations:** 1 Biophysics and Nuclear Medicine, University Hospitals of Strasbourg, 67098 Strasbourg, France; mehdi.st.helali@gmail.com (M.H.); celine.heimburger@chru-strasbourg.fr (C.H.); caroline.bund@chru-strasbourg.fr (C.B.); Fabrice.HUBELE@chru-strasbourg.fr (F.H.); 2 Radiophysics, Centre Paul-Strauss, UNICANCER, 67065 Strasbourg, France; MMoreau@strasbourg.unicancer.fr; 3 Radiotherapy, Centre Paul-Strauss, 67065 Strasbourg, France; c-lefevre56@hotmail.fr (C.L.F.); gnoel@strasbourg.unicancer.fr (G.N.); 4 ICube, University of Strasbourg/CNRS (UMR 7357) and FMTS, Faculty of Medicine, 67000 Strasbourg, France; 5 Internal Medicine, University Hospitals of Strasbourg, Strasbourg University, 67098 Strasbourg, France; Bernard.Goichot@chru-strasbourg.fr; 6 Radiology, University Hospitals of Strasbourg, Strasbourg University, 67098 Strasbourg, France; Francis.Veillon@chru-strasbourg.fr; 7 Otolaryngology and Maxillofacial Surgery, University Hospitals of Strasbourg, 67098 Strasbourg, France; Anne.Charpiot@chru-strasbourg.fr; 8 Université de Strasbourg, CNRS, IPHC UMR 7178, Centre Paul Strauss, UNICANCER, 67065 Strasbourg, France

**Keywords:** paraganglioma, head and neck, radiotherapy, ^18^F-FDOPA, PET, GTV

## Abstract

In this simulation study, we assessed differences in gross tumor volume (GTV) in a series of skull base paragangliomas (SBPGLs) using magnetic resonance imaging (MRI), ^18^F-dihydroxyphenylalanine (^18^F-FDOPA) combined positron emission tomography/computed tomography (PET/CT), and ^18^F-FDOPA PET/MRI images obtained by rigid alignment of PET and MRI. GTV was delineated in 16 patients with SBPGLs on MRI (GTV_MRI_), ^18^F-FDOPA PET/CT (GTV_PET_), and combined PET/MRI (GTV_PET/MRI_). GTV_PET/MRI_ was the union of GTV_MRI_ and GTV_PET_ after visual adjustment. Three observers delineated GTV_MRI_ and GTV_PET/MRI_ independently. Excellent interobserver reproducibility was found for both GTV_MRI_ and GTV_PET/MRI_. GTV_PET_ and GTV_MRI_ were not significantly different. However, there was some spatial difference between the locations of GTV_MRI_, GTV_PET_, and GTV_PET/MRI_. The Dice similarity coefficient median value was 0.4 between PET/CT and MRI, and 0.8 between MRI and PET/MRI. The combined use of PET/MRI produced a larger GTV than MRI alone. Nevertheless, both the target-delivered dose and organs-at-risk conservancy were respected when treatment was planned on the PET/MRI-matched data set. Future integration of ^18^F-FDOPA PET/CT into clinical practice will be necessary to evaluate the influence of this diagnostic modality on SBPGL therapeutic management. If the clinical utility of ^18^F-FDOPA PET/CT and/or PET/MRI is confirmed, GTV_PET/MRI_ should be considered for tailored radiotherapy planning in patients with SBPGL.

## 1. Introduction

Head and neck paragangliomas (HNPGLs) are rare and slow-growing tumors that result from paraganglia, neural crest-derived clusters of neuroendocrine cells. HNPGLs account for about 70% of extra-adrenal PGLs and develop from parasympathetic paraganglia of the jugular bulb and carotid body, or along the tympanic branch of the glossopharyngeal nerve, the vagus nerve, and its auricular branch [1]. About a third of HNPGLs are hereditary, mostly related to the mutation of the succinate dehydrogenase (*SDH*) complex genes [2]. When malignant, HNPGLs generally spread into the regional lymph nodes, lung, and bone [3]. Magnetic resonance imaging (MRI) and MR angiography are very accurate for tumor detection and local extension definition [4]. Combined positron emission tomography and computed tomography (PET/CT) with ^18^F-dihydroxyphenylalanine (^18^F-FDOPA) is highly sensitive (91%) and specific (95%) and is currently proposed as the first-line nuclear imaging modality in HNPGLs both at staging and during the post-treatment follow-up [5,6,7]. Once internalized, ^18^F-FDOPA is decarboxylated to ^18^F-dopamine, transported and stored in secretory vesicles. Indirectly, in PGLs, ^18^F-FDOPA uptake reflects the pathological up-regulation of the catecholamine biosynthetic pathway [8].

Treatment of HNPGLs is often personalized and influenced by genetic status, lesion size and location, tumoral multifocality, patient age, and comorbidities [9]. Radiotherapy and stereotactic radiotherapy are proposed as valuable therapeutic options for HNPGLs as they are less invasive than surgery, especially for patients with skull-base paragangliomas (SBPGLs) [10,11,12,13]. Due to continuing technological advances, the role of such treatments has increased progressively in the last few decades, achieving excellent rates of local tumor control and patient outcome with few iatrogenic effects [14]. It is important to underline that PGLs are frequently characterized by slow cellular turnover rates, efficient DNA repair mechanisms, and consequently low radiosensitivity, requiring an elevated radiation dose to overcome radioresistance. On the other hand, the presence of surrounding critical neuroanatomical structures is an important factor to be taken into account, usually limiting the tumor’s delivered dose [13]. The definition of gross tumor volume (GTV) is the first step of primary importance in planning external radiation therapy and is strictly related to the final irradiated volume. In the last few decades, continuous and successful technical improvements for external radiotherapy treatment have been seen, leading to the development of highly conformal intensity-modulated radiation therapy (IMRT) and personalized irradiation approaches. Consequently, remarkable efforts have been made to optimize GTV delineation. The current availability of hybrid multimodality imaging is gradually changing the paradigm for radiotherapy planning definition, which is classically based on CT or MRI imaging. Tumor morphological definition and functional characterization, combined in a single diagnostic exploration (i.e., PET/CT), could improve the definition of both GTV, which will receive the highest dose, and clinical target volume (CTV), which includes the subclinical tumor extension not visible on imaging modalities and subjective for many locations. Moreover, the recent availability of PET/MRI devices offers the potential advantages of high soft-tissue contrast and functional MRI capability to improve the diagnosis of cancer and its phenotype characterization. Several authors showed that the combination of MRI and PET potentially improves the accuracy of both the primary tumor and metastatic lymph node delineation in patients with HN malignancies, with consequent clinical advantages in disease control and toxicity reduction [15,16]. At present, radiotherapy planning for HNPGLs is defined utilizing contrast-enhanced CT and/or MRI. Metabolic information provided by PET/CT is only sporadically integrated. Moreover, no definitive consensus has been reached on the optimal modality for GTV definition on ^18^F-FDOPA PET/CT in patients with HNPGLs. On the other hand, semi-quantitative uptake parameters such as the tumor-to-brain ratio (TBR) were successfully used to delineate gliomas on PET imaging with radiolabeled amino acids [17,18,19,20,21]. Overall, despite potential diagnostic advantages related to functional imaging [22], to our knowledge there are no reports concerning the use of ^18^F-FDOPA PET/CT to delineate target volumes in patients with HNPGLs. In view of the above, the purpose of this simulation study was to assess the differences in GTV using contrast-enhanced MRI, ^18^F-FDOPA PET/CT, and combined PET/MRI images in a series of SBPGLs. We also evaluated the safety of irradiation therapy using PET/MRI fusion images, and in selected patients, compared the radiation treatment planning and dosimetry obtained from GTV assessed by MRI, which is the standard at several institutions, and PET/MRI-registered images.

## 2. Results

### 2.1. Patients

Sixteen consecutive patients with jugulotympanic SBPGLs were retrospectively included (nine men and seven women, mean age: 57 years, range: 37–84 years). Patient characteristics are detailed in Table 1. Seven and nine patients were evaluated at primary staging and during follow-up, respectively, because of clinical suspicion of tumor recurrence. Previous treatment included surgery, radiotherapy, and ^90^Y-DOTATOC peptide receptor radionuclide therapy in seven, three, and three patients, respectively. Two patients were succinate dehydrogenase subunit B (*SDHB*) and *SDHC* mutation carriers. In the remaining 14 cases, the PGLs were apparently sporadic. No patient presented with regional lymph nodes or systemic metastases at the time of diagnostic imaging.

### 2.2. Tumor Volume Assessment

• MRI

The median lesion size was 17 mm (range: 7–40 mm). The median values of GTV_MRI_ were 1.4 cm^3^ (range: 0.2–8.6 cm^3^), 1.7 cm^3^ (range: 0.3–9.6 cm^3^), and 1.2 cm^3^ (range: 0.2–8.7 cm^3^) for the three observers. According to intraclass correlation coefficient (ICC) analysis, MRI was a highly reproducible method for GTV delineation (agreement coefficient: 0.95). The GTV_MRI_ assessed by the most experienced radiation oncologist (observer 1) was considered for the definition of both CTV_MRI_ (median value: 26.2 cm^3^; range: 11.9–51.2 cm^3^), and planning target volume (PTV_MRI_) (median value: 54.1 cm^3^; range: 21.8–79.1 cm^3^).

• ^18^F-FDOPA PET/CT and PET/MRI

**Figure 1:**
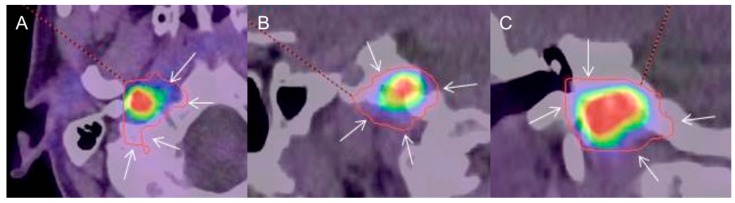
Typical example of gross tumor volume assessed by tumor-to-brain ratio (GTV_TBR_) (red contour) evaluated on ^18^F-dihydroxyphenylalanine (^18^F-FDOPA) combined positron emission tomography/computed tomography (PET/CT) images (**A**: axial, **B**: sagittal, **C**: coronal) in a patient with a sporadic right skull base paraganglioma (SBPGL). A threshold value of 1.6 over the background uptake was used as the reference for semi-automatic definition of GTV. Note that GTV_TBR_ largely exceeds the metabolic tumoral edges (arrows).

Despite substantial heterogeneity of tumoral ^18^F-FDOPA uptake among the patients studied, SBPGLs were distinctly detectable by PET/CT in all cases (median value of maximum standardized uptake value (SUV_max_): 11.5; range: 1.4–82.8). The median value of GTV_PET_ was 0.9 cm^3^ (range: 0.3–17.1 cm^3^). In this series, although GTV_PET_ was lower than GTV_MRI_, no significant difference was assessed when considering the entire population (*p* = 0.09), or only previously treated patients (*p* = 0.12), or only treatment-naïve patients (*p* = 1). GTV_TBR_ (median value: 5.1 cm^3^; range: 0.3–26.1 cm^3^) was significantly larger than both GTV_PET_ (*p* = 0.01) and GTV_MRI_ (*p* = 0.006). GTV_TBR_ largely exceeded tumoral boundaries on PET images, including several extratumoral voxels (with a similar activity to that of the background), often protruding in apparently healthy bone structures and an adjacent vasculonervous pedicle (Figure 1). Therefore, GTV_TBR_ has not been further considered and the GTV_PET/MRI_ was assessed combining GTV_MRI_ and GTV_PET_ after visual adjustment by the radiation oncologist. The median values of GTV_PET/MRI_ were 3.2 cm^3^ (range: 0.5–18.8 cm^3^), 2.7 cm^3^ (range: 0.3–11.8 cm^3^), and 2.3 cm^3^ (range: 0.4–17.1 cm^3^) for the three observers. ICC analysis showed excellent interobserver reproducibility with an agreement coefficient of 0.91. GTV_PET/MRI_ assessed by the most experienced radiation therapist (observer 1) was used to estimate both CTV_PET/MRI_ (median value: 33.8 cm^3^; range: 15–82.7 cm^3^) and PTV_PET/MRI_ (median value: 62.7 cm^3^; range: 26.5–112.7 cm^3^). GTV_PET/MRI_, CTV_PET/MRI_, and PTV_PET/MRI_ were significantly larger than GTV_MRI_ (*p* = 0.00003), CTV_MRI_ (*p* = 0.003), and PTV_MRI_ (*p* = 0.003), respectively. The details of GTV comparison between MRI and PET/CT, and between MRI and PET/MRI are reported in Table 2.

### 2.3. Positional GTV Assessment

**Figure 2:**
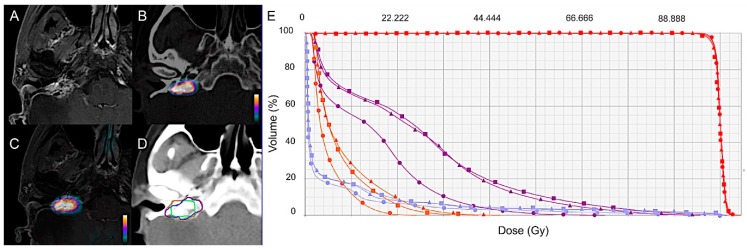
GTV delineation and dose-volume histogram (DVH) based on MRI (Δ), ^18^F-FDOPA PET/CT (●), and ^18^F-FDOPA PET/MRI (■) for a representative case. Contrast-enhanced T1-weighted MRI (**A**), ^18^F-FDOPA PET/CT (**B**), and combined PET/MRI (**C**) axial images in a 57-year-old woman with a relapsing 23-mm right jugulotympanic *SDHC* PGL previously treated with surgery and peptide receptor radionuclide therapy (patient 2, Table 1). (**D**) GTV delineation for external radiation therapy based on MRI (blue contour), ^18^F-FDOPA PET/CT (green contour), and combined ^18^F-FDOPA PET/MRI images (orange contour). (**E**) Radiation treatment planning based on MRI, ^18^F-FDOPA PET/CT, and combined ^18^F-FDOPA PET and MRI data set assessed for volumetric-modulated arc therapy. Dose-volume histogram for PTV and organs at risk (OAR) are displayed. PTV: red curves; brainstem: purple curves; mandible: blue curves; parotid: orange curves. All the treatment plans were able to respect clinical objectives showing similar results concerning both target delivered dose and OAR conservancy.

**Figure 3:**
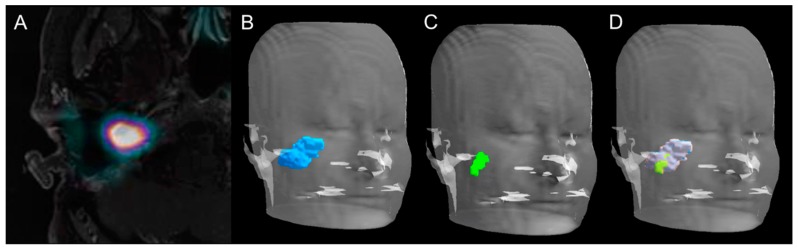
Volume rendering technique representations of GTV delineated on PET, MRI, and PET/MRI. Axial slice of combined ^18^F-FDOPA PET/MRI imaging in a 43-year-old woman (patient 5, Table 1) with a relapsing 40-mm apparently sporadic right jugulotympanic PGL previously treated with surgery (**A**). Volume rendering technique representation of GTV delineated on MRI (**B**, blue volume), ^18^F-FDOPA PET/CT (**C**, green volume), and combined ^18^F-FDOPA PET/MRI imaging (**D**, green and gray volume).

There was some spatial difference between the locations of GTV delineated on MRI, PET/CT, and PET/MRI (Figure 2 and Figure 3). According to the analysis of positional variability of GTVs, the median intersection volume between GTV_PET_ and GTV_MRI_ was 0.6 cm^3^ (range: 0.1–6.0 cm^3^), and between GTV_MRI_ and GTV_PET/MRI_ it was 1.5 cm^3^ (range: 0.3–9.3 cm^3^). The median DSC was 0.4 (range: 0.1–0.8) between PET and MRI, and 0.8 (range: 0.4–1) between MRI and PET/MRI. The intersection volume between GTV_PET_ and GTV_MRI_ correlated positively with the size of the lesion (R = 0.84, *p* = 0.0001) and was significantly lower (*p* = 0.04) in patients with relapsing tumor (median: 0.9 cm^3^; range: 0.3–12.1 cm^3^) compared to newly diagnosed patients (median: 2.4 cm^3^; range: 1.2–8.8 cm^3^). Table 2 summarizes the results of positional GTV analysis and the DSC index.

Uniform expansions of the GTV_MRI_ contours were performed in increments of 1 mm until 100% of the GTV_PET/MRI_ was covered. An average expansion of 7 mm (median: 7 mm; range: 2–9 mm) beyond contrast-enhanced T1-weighted MRI contours was necessary to cover 100% of the ^18^F-FDOPA PET/MRI primary tumor volume. Finally, a mean contraction of 3.6 mm (median: 3 mm; range: 1–8 mm) of CTV_MRI_ made it possible to encompass the GTV_PET/MRI_.

### 2.4. Radiation Treatment Planning

MRI and PET/MRI-based radiation treatment planning was assessed in three patients with apparently sporadic relapsing jugulotympanic PGLs (Table 1, cases 2, 3, and 5). Patients were selected according to tumor size aiming to simulate treatment for tumors of different sizes, ranging from a few millimeters to several centimeters. Detailed results of the dosimetric evaluation in each patient are listed in Table 3 and Table 4. Overall, all the plans generated on ^18^F-FDOPA PET/MRI were able to respect clinical objectives despite the size discrepancy existing between GTV_MRI_ and GTV_PET/MRI_. PTV_MRI_ and PTV_PET/MRI_ plans were similar concerning both target delivered dose and OAR conservancy (deviation under 2.7% of prescription dose and 0.6% of OAR volume for dose and volume, respectively) without underdosing of GTV_PET/MRI_ compared to target volumes generated on treatment plans using MRI alone (Table 3, Figure 3). Dosimetric details concerning tumoral target and OAR are reported in Table 4.

## 3. Discussion

To our knowledge, the present study evaluates for the first time the differences in GTV delineation using contrast-enhanced MRI, ^18^F-FDOPA PET/CT, and combined PET/MRI images in patients with SBPGLs. We also compared the radiation treatment planning based on the MRI and PET/MRI data set, suggesting the safety of irradiation therapy using PET/MRI fusion images showing no differences in tumor-delivered dose and OAR conservancy between MRI- and PET/MRI-based radiation planning, regardless of the intermodality degree of volumetric agreement.

Although there were individual cases with greater volumetric disparities, GTV_PET_ and GTV_MRI_ were not significantly different. However, a trend toward significance was observed when considering the entire patient cohort, and the lack of statistical significance might be due to the limited number of patients studied. GTV_PET/MRI_, which was defined as the union of GTV_MRI_ and GTV_PET_ after adjustment by a radiation oncologist, encompasses nearly all GTV regions. An average expansion of 7 mm beyond the MRI T1-gadolinum contours allowed 100% coverage of the ^18^F-FDOPA PET/MRI volumes. Accordingly, as expected, the combined use of PET/MRI produced a larger GTV than MRI alone. In spite of this, both target delivered dose and OAR conservancy were respected when PTV was planned on MRI or the PET/MRI matched data set, probably due to the slight positional discordance as shown by the good DSC average value (0.8). V95 (i.e.: volume of PTV receiving 95% of prescription) was near the prescribed dose and was not significantly different between MRI- and PET/MRI-based GTVs for each patient and on average.

The integration of ^18^F-FDOPA PET/CT to MRI was evaluated for radiotherapy planning of gliomas. In these patients, ^18^F-FDOPA PET/CT generated larger target volumes compared to the standard-of-care MRI. The result was a customization of radiotherapy plans by the inclusion of “metabolic disease” without contrast enhancement [23,24,25]. Navarria et al. emphasized the idea of “biologic tumor volume” in a population of 69 patients with high-grade gliomas [26]. They showed that 50% of radiotherapy failures occurred outside the contrast-enhanced volume on T1-weighted MRI sequences and would have been included within the target volume generated according to ^11^C-methionine PET.

DSC analysis revealed incongruences of GTV position between MRI and ^18^F-FDOPA PET/CT in patients with SBPGL, warranting further investigations, longitudinal patient follow-up, and histopathology correlation. Overlap differences between GTV_MRI_ and GTV_PET_ could be attributable to several factors and needs to be discussed. First of all, no hybrid PET/MRI device was used for patient exploration. Indeed, for GTV delineation, the PET/CT and MRI data set were matched using a semi-automated volume-based registration algorithm with consequent potential spatial uncertainty in target volume identification induced by image misalignment. Secondly, a semi-automated SUV_max_-based segmentation algorithm was used to outline the target volume on PET/CT images, taking into account the value of 40% of SUV_max_ according to ^18^F-Fluorodeoxyglucose (^18^F-FDG) PET/CT-related literature in general oncology. As reported, automated methods of image segmentation would be preferable to determine the metabolically active tumor volume (MATV) [27]. MATV delineation based on semi-quantitative uptake parameters such as the TBR has long been used for PET imaging of brain tumors with radiolabeled amino acids. In a biopsy-controlled study using ^18^F-Fluoroethyl-l-tyrosine (^18^F-FET) PET in patients with brain tumors [17], a threshold value of 1.6 over the background uptake was taken as the reference for a semi-automatic definition of tumor volume (GTV_TBR_). Based on the assumption that the TBR contrast of ^18^F-FDOPA uptake in brain tumor is similar to that of ^18^F-FET [20,21], other authors successfully adopted the same approach in patients with gliomas investigated by ^18^F-FDOPA PET/CT [18,19]. In our patients, GTV_TBR_ assessed in a similar manner was significantly larger than that obtained using 40% of tumor SUVmax or MRI. Moreover, GTV_TBR_ largely exceeded tumoral limits on PET images, covering apparently healthy bone or adjacent vasculonervous structures (Figure 1). Therefore, both the lack of biopsy-proven evidence of tumoral invasion (contrary to what Pauleit et al. have proven in gliomas [17]) and the potential high risk of radiation after-effects require further investigation before clinical utilization of this type of delineation method for SBPGLs. The heterogeneity of tumor size, ranging from 7 mm to 40 mm, and the high inter-tumor variability of ^18^F-FDOPA uptake (range: 1.4–82.8) could contribute to explaining the difficulty in properly defining GTV_TBR_, especially for lesions with metabolic activity as high as PGLs. Interestingly, in our population, the overall mean TBR was 16.3 (range: 1.4–83.4), approximately nine-fold higher than the TBR reported for brain tumors on ^18^F-FDOPA PET studies (1.76 ± 0.60) [18]. Finally, optimal ^18^F-FDOPA PET/CT segmentation algorithms for SBPGL GTV_PET_ contouring need to be optimized, also taking into account the lessons learned from patients with gliomas.

The third and last point to discuss concerns the population studied for GTV delineation, including patients naïve of treatment and subjects with relapsing tumors after surgery and/or radiotherapy. The iatrogenic distortion of regional anatomic architecture may lead to modification of vascular patterns and tissue enhancement on CT and MRI studies. Contrast medium arrival during the early arterial phases could be delayed and less pronounced, leading to erroneous image interpretations in patients with relapsing local disease [7,28]. Interestingly, overlap differences between GTV_PET_ and GTV_MRI_ were more pronounced for relapsing tumor compared to newly diagnosed lesions. In those patients, an average 46% of the ^18^F-FDOPA PET/CT target volume extended outside GTV_MRI_ showing no pathological gadolinium enhancement. Integration of PET to MRI data could be advantageous for GTV delineation in previously treated patients due to a potentially challenging definition of tumoral infiltration [7]. In view of the above, the availability of PET/MRI hybrid devices will lead to radiotherapy planning based on spatially and temporally registered morphofunctional images [29].

It is important to underline that the majority of patients included in the present study presented with relatively small, benign and sporadic SBPGLs and that even the two cases of *SDH*-related PGLs did not have regional lymph node metastases. Consequently, to confirm our preliminary results, additional studies are required including patients with more aggressive and locally advanced tumors, in which modifications of GTV could have an important dosimetric impact and possibly clinical consequences. An additional attractive axis of clinical research, which would advance a further step towards the transition from the morphological tumor volume to the morphofunctional tumor volume concept, could be the comparison of ^18^F-FDOPA, ^18^F-FDG, and ^68^Ga-DOTA-peptides for PET-based GTV delineation. 

In a real clinical scenario, one more point of volumetric uncertainty is the definition of CTV usually made on CT or MRI. Ligtenberg et al. [30] recently determined the modality-specific CTV margins for CT, MRI, and ^18^F-FDG PET in patients with laryngohypopharyngeal tumors. Although GTV overestimated the tumor volume in all modalities, CTV margins were needed to achieve complete tumor delineation. Interestingly, PET-based CTVs were the smallest and considered to be the most accurate, while MRI-based CTVs were larger than PET- and CT-based CTVs. In our patient population, by a mean contraction of 3.6 mm (median: 3 mm; range: 1–8 mm) of CTV_MRI_, we encompassed every GTV_PET/MRI_. This observation could contribute to the debate regarding the choice of CTV threshold, suggesting a role of hybrid PET/MRI imaging to modulate CTV margin expansion tailored to each clinical situation. It is possible that increased accuracy in GTV delineation with PET/MRI could allow the application of smaller CTV margins, possibly reducing toxicity while conserving reliability in tumor coverage and treatment efficacy.

Another advantage of multimodality imaging is likely the ability to specify a volume at high risk of relapse, which could be better controlled by the use of simultaneous integrated boost (SIB). This method has already been used in several tumors without deterioration of OAR protection [31,32].

## 4. Materials and Methods

### 4.1. Patients

The medical records of patients with clinical, radiological, and/or pathological diagnosis of HNPGLs referred to the Nuclear Medicine Department of Strasbourg University Hospitals from May 2012 to April 2017 for ^18^F-FDOPA PET/CT were retrospectively reviewed. Only patients with confirmed SBPGLs, with positive ^18^F-FDOPA PET/CT findings, and who underwent MRI within less than 3 months of ^18^F-FDOPA PET/CT were retrospectively included. Conversely, patients without a final diagnosis of PGL, or patients with PGLs not arising from the skull base, or patients for whom MRI data were not fully available were not selected for the study.

Consistent with local institutional guidelines, all patients included gave free and informed consent for the use of anonymous personal medical data extracted from their file for scientific purposes. The local institutional review board approved this retrospective study (FC/dossier 2018-49).

### 4.2. Reference Diagnostic Imaging

HN MRI investigations were performed with a 1.5-T (Avanto, Siemens, Medical Systems, Erlangen, Germany) or a 3-T (Signa, General Electric Medical System, Milwaukee, WI, USA) scanner. Morphological T1-weighted contrast-enhanced axial and coronal images with a 1-mm slice thickness were used for diagnostic purposes and for radiotherapy volume delineation. ^18^F-FDOPA PET/CT scans were performed using a combined PET/CT equipped by time of flight measurement capacity (Biograph mCT, Siemens Medical Systems, Erlangen, Germany). ^18^F-FDOPA was used in the setting of approved marketing authorization. Patients fasted for at least 4 h before tracer injection. In all patients, 4 MBq/kg of ^18^F-FDOPA was intravenously injected without carbidopa premedication. Whole-body ^18^F-FDOPA PET/CT acquisition was performed about 30 min after radiotracer injection from the top of the skull to the upper thigh (4 min per step) starting from the head. CT studies for attenuation correction and anatomic registration were performed without administration of contrast medium. PET data were reconstructed iteratively. CT, PET (after attenuation correction), and PET/CT images were displayed on a dedicated workstation for analysis. A focal area of increased ^18^F-FDOPA uptake in a usual anatomical site for paraganglia was considered as a positive finding. The tumor maximum standardized uptake value (SUV_max_) was defined within a spherical volume of interest (VOI) centered on the tumor and including it completely. To obtain PET/MRI images, ^18^F-FDOPA PET/CT were matched and registered with T1-weighted contrast-enhanced MRI including the whole SBPGL. MRI sequences were rigidly aligned to the CT data set of PET/CT using a semi-automated volume-based registration algorithm (Focal software, CMS-XIO).

### 4.3. Tumor Volume Assessment

Tumor volumes and radiation plans based on ^18^F-FDOPA PET/CT, MRI, and matched PET/MRI images were delineated for research purposes only and were not used prospectively for radiation treatment planning in any patient. GTV encompasses the recognizable macroscopic tumor infiltration and defines both the extent and position of the primary tumor. In our series, GTV was assessed on the MRI (GTV_MRI_), ^18^F-FDOPA PET/CT (GTV_PET_), and matched PET/MRI data set (GTV_PET/MRI_). Two experienced radiation oncologists (observers 1 and 2) and one nuclear medicine physician (observer 3) independently performed the GTV delineation on MRI and fused PET/MRI data while aware of patient clinical history. To prevent biases, ^18^F-FDOPA PET/CT results were not available before GTV_MRI_ definition. Similarly, GTV_MRI_ data were not accessible before GTV_PET/MRI_ delineation. GTV_MRI_ was delineated using axial contrast-enhanced T1-weighted MRI images. GTV_PET_ was assessed on axial images using the automatic assistant arbitrarily calibrated at 40% of SUV_max_ of the primary tumor (Syngo.via VB10B, Siemens) according to the ^18^F-FDG PET/CT-related literature in clinical oncology [33]. To delineate SBPGL on PET/CT imaging, the tumor-to-brain ratio (TBR) was also used. Based on previous studies on gliomas [17,18,19,20,21], a threshold value of 1.6 over the background uptake was taken as the reference for a semi-automatic definition of tumor volume (GTV_TBR_). To measure background activity, a large region of interest above the upper ventricle and including both gray and white matter was used. Lastly, GTV_PET/MRI_ was the union of GTV_MRI_ and GTV_PET_ and the final contour assessment was made based on visual adjustment of the images by the treating radiation oncologist. MRI- and PET/CT-related GTV and intersection volume were assessed using ARTIVIEW™ software (AQUILAB^®^, Lille, France). Concordance between GTV_MRI_ and GTV_PET_ contours and between GTV_MRI_ and GTV_PET/MRI_ contours was evaluated according to the Dice similarity coefficient (DSC), a validated index measuring spatial overlap between two volumes. The DSC was calculated as follows: 2 × (A ∩ B)/(A + B), where A and B represent two volumes, (A ∩ B) represents the volume of intersection, and (A + B) represents the sum of their volumes. A DSC ≥ 0.7 can be considered as a “good” overlap [34]. According to the standard of care of our institution, CTV and PTV of patients were defined by adding a 10-mm three-dimensional (3D) margin to GTV and a 3-mm 3D margin to CTV, respectively. Hence, starting from GTV, we assessed CTV and PTV on the MRI (CTV_MRI_, PTV_MRI_), ^18^F-FDOPA PET/CT (CTV_PET_, PTV_PET_), and matched PET/MRI data set (CTV_PET/MRI_, PTV_PET/MRI_).

### 4.4. Radiation Treatment Planning

Dosimetric evaluation was performed for research purposes only aiming to assess the potential consequence of any volumetric differences between gold standard MRI and new PET/MRI-related GTV. In other words, we researched the eventual reduction of tumoral dose delivered when metabolic GTV_PET_ data were combined with GTV_MRI_. Complete radiation treatment planning was assessed for volumetric-modulated arc therapy (VMAT). VMAT is a technique for IMRT that simultaneously combines varying dose rate, gantry speed, and the shape of the multileaf collimator aperture [35]. VMAT plans were generated using the Eclipse treatment planning system (version 11.0.31, Varian Medical Systems) on for a delivered dose of 45 Gy with 1.8-Gy fractions. The dose calculation was performed with the AAA algorithm and a 2.5-mm grid size; for the optimization, the PRO3 algorithm was used. Two co-planar double-arcs were generated for each PTV, where the collimator angle was set to 30° for counter-clockwise rotation and 330° for clockwise rotation. During the first optimizing process, the objectives (PTV and OAR) were the same for the two plans. During a second optimizing process, the penalties (dose-volume objective and/or weight) corresponding to the OAR were manually adapted to minimize the absorbed dose according to the clinical objectives. Dose to PTV was optimized and normalized to obtain the same V95% (volume of PTV receiving 95% of prescription dose), D98% (dose received by 98% of the PTV) and D2% (dose received by 2% of the PTV), with a maximum deviation of 1%. Radiotherapy planning was based on the MRI and PET/MRI GTV data set adding a standard 10-mm 3D margin to GTV to obtain CTV, and a 3-mm 3D margin to CTV for PTV definition.

### 4.5. Statistical Analysis

The results for continuous data were expressed as median and range, whereas categorical variables were presented as numbers and percentages. GTV, CTV, and PTV data were expressed in cubic centimeters. The intraclass correlation coefficient (ICC) was used for interobserver reproducibility assessment of both GTV_MRI_ and GTV_PET/MRI_. ICC inter-rater agreement measurements were interpreted according to the following criteria: less than 0.40 = poor agreement; 0.40–0.59 = fair agreement; 0.60–0.74 = good agreement; 0.75–1 = excellent agreement [36]. A two-way mixed effect model with absolute agreement definition parameters was applied to the ICC. The nonparametric paired-sample Wilcoxon signed-rank test was used to evaluate differences between GTV_MRI_ and GTV_PET_, and between GTV_MRI_ and GTV_PET/MRI_, and to compare doses delivered according to GTV obtained from MRI and PET/MRI data. The nonparametric Mann–Whitney *U* test was used to test differences between patient groups. The Spearman rank correlation test was conducted to assess the relationship between variables. All statistical analyses were performed using R Studio (Version 1.0.153 2017, R Studio, Inc., Boston, MA, USA). A *p*-value less than 0.05 was considered significant.

## 5. Conclusions

In this era of multimodality imaging, we should consider that no single imaging modality encompasses an entire microscopic and macroscopic tumor, pointing out the difficulty selecting which imaging modality is superior for target volume delineation. In our opinion, the real question that at present remains without a definitive response is whether any difference in GTV delineation according to the available imaging modalities (MRI, PET/CT, and PET/MRI) is clinically significant in patients with SBPGL. For the moment, we can note that no differences exist in terms of tumor-delivered dose between MRI and PET/MRI-based radiation planning, regardless of the inter-modality degree of volumetric agreement. Future integration of ^18^F-FDOPA PET/CT into clinical practice for SBPGL radiotherapy planning will be necessary to evaluate the influence of this combined diagnostic modality on tumor eradication or local control. If the clinical utility of ^18^F-FDOPA PET/MRI is further confirmed in a large patient population, combined GTV_PET/MRI_ should be considered for tailored SBPGL radiotherapy planning to identify positive disease not clearly detected by conventional MRI, to redefine CTV margins, or to give a radiation boost treatment.

## Figures and Tables

**Table 1:** Patient population characteristics.

Patient	Age, Sex (Man/Woman)	Symptoms	PGL Size (mm), Side (Left/Right)	Genetics	Primary Staging, Recurrence	Prior Treatment
1	62, M	Tinnitus	10, L	Sporadic	Primary Staging	-
2	57, W	Tinnitus Ear discharge	23, R	*SDHC* ^3^	Recurrence	Surgery PRRT ^2^
3	57, M	Tinnitus	8, R	Sporadic	Recurrence	Surgery
4	58, M	Tinnitus	8, R	Sporadic	Recurrence	Surgery IMRT ^3^
5	43, W	Tinnitus Local pain	40, R	Sporadic	Recurrence	Surgery
6	84, W	Tinnitus	31, L	Sporadic	Recurrence	Surgery Gamma Knife
7	62, W	Asymptomatic	22, L	*SDHB* ^1^	Recurrence	Surgery
8	67, W	Pulsatile tinnitus	8, L	Sporadic	Primary Staging	-
9	66, M	Dizziness	25, R	Sporadic	Primary Staging	-
10	70, W	Pulsatile tinnitus	21, L	Sporadic	Recurrence	PRRT ^2^
11	47, M	Pulsatile tinnitus	7, L	Sporadic	Primary Staging	-
12	48, M	Pulsatile tinnitus	13, L	Sporadic	Recurrence	IMRT ^3^
13	59, W	Asymptomatic	22, R	Sporadic	Recurrence	Surgery PRRT ^2^
14	54, M	Tinnitus Ear discharge	12, L	Sporadic	Primary Staging	-
15	58, M	Pulsatile tinnitus	9, R	Sporadic	Primary Staging	-
16	37, M	Dizziness Hearing loss	30, R	Sporadic	Primary Staging	-

^1^*SDHB*: succinate dehydrogenase subunit B; ^2^ PRRT = peptide receptor tadionuclide therapy; ^3^ IMRT: intensity-modulated radiotherapy.

**Table 2:** Volumetric and positional analysis of GTVs assessed by magnetic resonance imaging (MRI), ^18^F-FDOPA PET/CT, and PET/MRI.

Patient	GTV ^a^ (cm^3^)			DSC ^b^	
	MRI	PET/CT	PET/MRI	MRI vs. PET/CT	MRI vs. PET/MRI
1	0.33	0.63	0.87	0.51	0.65
2	4.01	2.23	5.17	0.59	0.95
3	1.25	0.28	1.60	0.30	0.99
4	1.35	0.49	1.85	0.39	0.82
5	5.95	3.66	9.95	0.53	0.43
6	4.93	4.73	6.84	0.76	0.89
7	8.58	1.40	10.46	0.21	0.97
8	0.19	0.29	0.44	0.58	0.72
9	6.01	17.10	18.76	0.52	0.52
10	1.44	2.06	3.08	0.53	0.73
11	1.42	0.83	3.25	0.06	0.62
12	1.35	0.84	2.68	0.14	0.66
13	4.10	0.92	4.85	0.33	0.99
14	0.76	0.83	1.64	0.33	0.74
15	0.55	0.62	1.20	0.39	0.73
16	4.55	1.13	5.15	0.38	1.00
Median (range)	1.4 (0.19–8.58)	0.88 (0.28–17.1)	3.16 (0.44–18.76)	0.4 (0.06–0.76)	0.7 (0.43–1.0)
Mean (SD ^c^)	2.92 (2.54)	2.38 (4.12)	4.86 (4.79)	0.41 (0.18)	0.78 (0.18)

^a^ GTV: gross tumor volume. ^b^ DSC: Dice similarity coefficient. ^c^ SD: standard deviation.

**Table 3:** Comparison of dosimetric results obtained from MRI- and PET/MRI-based radiation treatment planning in three patients with apparently sporadic relapsing jugulotympanic PGLs (patients 2, 3, 5, Table 1).

Patient	PGL Size (mm) ^1^	V95% ^2^		D98% ^3^		D2% ^4^	
		MRI	PET/MRI	MRI	PET/MRI	MRI	PET/MRI
1	23	99.8	99.5	97.1	96.7	101.9	102.2
2	8	99.5	99	96.9	96.3	102.5	103.1
3	40	99.2	99.1	96.4	96.3	102.4	102.5

^1^ PGL size refers to MRI investigation; ^2^ V95%: volume of PTV (planning target volume) receiving 95% of prescription; ^3^ D98%: dose received by 98% of the PTV; ^4^ D2%: dose received by 2% of the PTV.

**Table 4:** Comparison of dosimetric results on organs at risk (OAR) obtained from MRI and PET/MRI-based radiation treatment planning in 3 patients with apparently sporadic relapsing jugulotympanic PGLs (patients no. 2, 3, and 5 of Table 1).

Table 1	SBPGL Size on MRI (mm) and Side (R/L ^2^)	OAR ^3^	D_max_^4^ (Gy)		D_mean_^5^ (Gy)		V15Gy ^6^ (%)	
			MRI	PET/MRI	MRI	PET/MRI	MRI	PET/MRI
Pt ^1^ 2	23/R	Brainstem	42.9	45.8	11.7	12.3		
		R Parotid	21.2	18.8	4.1	3.7	1.5	1
		R IAC ^7^	44.8	45.6	44.3	44.3		
		Mandible	45.6	45.3	3.1	3.3		
Pt 3	8/R	Brainstem	46.9	47.0	12.6	13.1		
		R Parotid	46.4	46.0	3.6	3.7	4.9	5.5
		L IAC *	2.5	2.8	2.2	2.7		
		L Cochlea *	3	3.3	2.3	2.5		
Pt 5	40/R	Brainstem	46.7	46.7	18.9	19.8		
		R Parotid	46.7	46.9	14.8	19.3	35.8	50.9
		R IAC	44.8	44.9	44.4	44.4		
		Mandible	45	45.8	2.6	3		

^1^ Pt: patient; ^2^ R: right; L: left; ^3^ OAR: organ at risk; ^4^ D_max_: dose maximum; ^5^ D_mean_: mean dose; ^6^ V15Gy: the volume receiving doses above 15 Gy; ^7^ IAC: internal auditory canal. * R IAC and R Cochlea are included in PTV.
